# Infectious Mononucleosis: An Uncommon Presentation of a Common Disease—a Case Report

**DOI:** 10.1002/ccr3.72222

**Published:** 2026-03-06

**Authors:** Mohammad Moini, Shiva Sheibani, Matin Ghazizadeh

**Affiliations:** ^1^ School of Medicine Tehran University of Medical Sciences Tehran Iran; ^2^ Department of Otolaryngology, Head and Neck Surgery, Taleghani Hospital Shahid Beheshti University of Medical Sciences Tehran Iran

**Keywords:** conservative treatment, Epstein–Barr virus infections, infectious mononucleosis, lymphadenopathy, pharyngitis

## Abstract

Infectious mononucleosis (IM), caused by the Epstein–Barr virus (EBV), typically presents with fever, pharyngitis, and lymphadenopathy. However, atypical presentations may occur, complicating the diagnostic process. A 19‐year‐old male presented with high‐grade episodic fever, cervical lymphadenopathy, and severe constitutional symptoms. The clinical presentation raised concern for serious underlying conditions due to prominent lymphadenopathy. Laboratory evaluation demonstrated marked lymphocytosis with atypical lymphocytes and positive EBV serology, confirming the diagnosis of IM. The patient was managed conservatively and achieved complete recovery on follow‐up. This case highlights the importance of including atypical IM in the differential diagnosis of patients presenting with fever and lymphadenopathy. Early recognition of IM may prevent misdiagnosis and avoid unnecessary invasive and costly investigations, reducing patient harm and health care burden.

## Key Clinical Message

In patients with fever and lymphadenopathy, failure to consider infectious mononucleosis among the infectious causes, particularly when pharyngitis is absent, may shift the diagnostic focus toward malignancy, leading to unnecessary, invasive, and costly investigations, such as bone marrow examination. Early recognition of atypical infectious mononucleosis is therefore essential to prevent patient harm and reduce avoidable health care burden.

## Graphical abstract

Atypical infectious mononucleosis without pharyngitis may mimic malignancy; early EBV testing enables accurate diagnosis and prevents unnecessary invasive investigations.

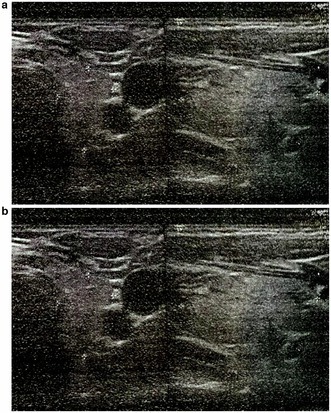

## 1 Introduction

Infectious mononucleosis (IM) is a clinical syndrome most commonly associated with Epstein–Barr virus (EBV), a member of the herpes family, while other pathogens, such as cytomegalovirus, may produce an IM‐like illness [1]. EBV is present in 90%–95% of adults, indicating that most of the world's population has been infected during childhood [2]. The life cycle of EBV consists of primary infection, lifelong latency, and lytic reactivation phases. Various cell types can become infected by EBV, but it mainly infects epithelial cells and B cells. Since one of the transmission ways is saliva, it is speculated that the first infected cells are mostly epithelial. When EBV virions are released from oropharyngeal epithelial cells, they reach the underlying tissue and infect B cells [3, 4].

Although EBV infection is asymptomatic in children or with mild pharyngitis, in adults it is mainly characterized by three symptoms: fever, tonsillar pharyngitis, and lymphadenopathy. In addition to the classic presentation, IM may occasionally manifest in a so‐called “typhoidal” form, characterized predominantly by prolonged fever and systemic constitutional symptoms with minimal or absent pharyngitis. Such presentations may closely resemble other systemic or malignant conditions, potentially complicating early diagnostic reasoning [5]. Other manifestations include debilitating fatigue, splenomegaly, the appearance of atypical lymphocytes in the peripheral blood, increased liver enzymes, rashes [1, 2], and systemic autoimmune diseases (SADs) [6]. It can also be oncogenic, which may result in several epithelial and lymphoid cell malignancies [7]. It can cause severe complications in less than 1% of cases [8]. Atypical cases of IM have been rarely reported so far and not being familiar with rare cases can result in inappropriate diagnosis and a wrong approach to the disease. Therefore, we report a case of IM presenting with prominent constitutional symptoms and lymphadenopathy, mimicking more serious conditions, to highlight an important diagnostic challenge in clinical practice. Written informed consent was obtained from the patient for participation and publication of this case report and the accompanying images.

## 2 Case History/Examination

A 19‐year‐old male was referred to the clinic with a chief complaint of severe shivers and a fever of 38.3 C°. The patient reported constitutional symptoms, including fatigue, and denied a sore throat or other symptoms suggestive of an upper respiratory or gastrointestinal infection. Upon physical examination, bilateral anterior cervical lymphadenopathy (more prominent on the left side) and mild splenomegaly were noted. There was no sign of pharyngitis, tonsillitis, or any exudate in the patient's oral cavity or pharynx. Also, no sign of upper and lower respiratory or gastrointestinal involvement was seen.

## 3 Differential Diagnosis/Investigations/Treatment

**FIGURE 1:**
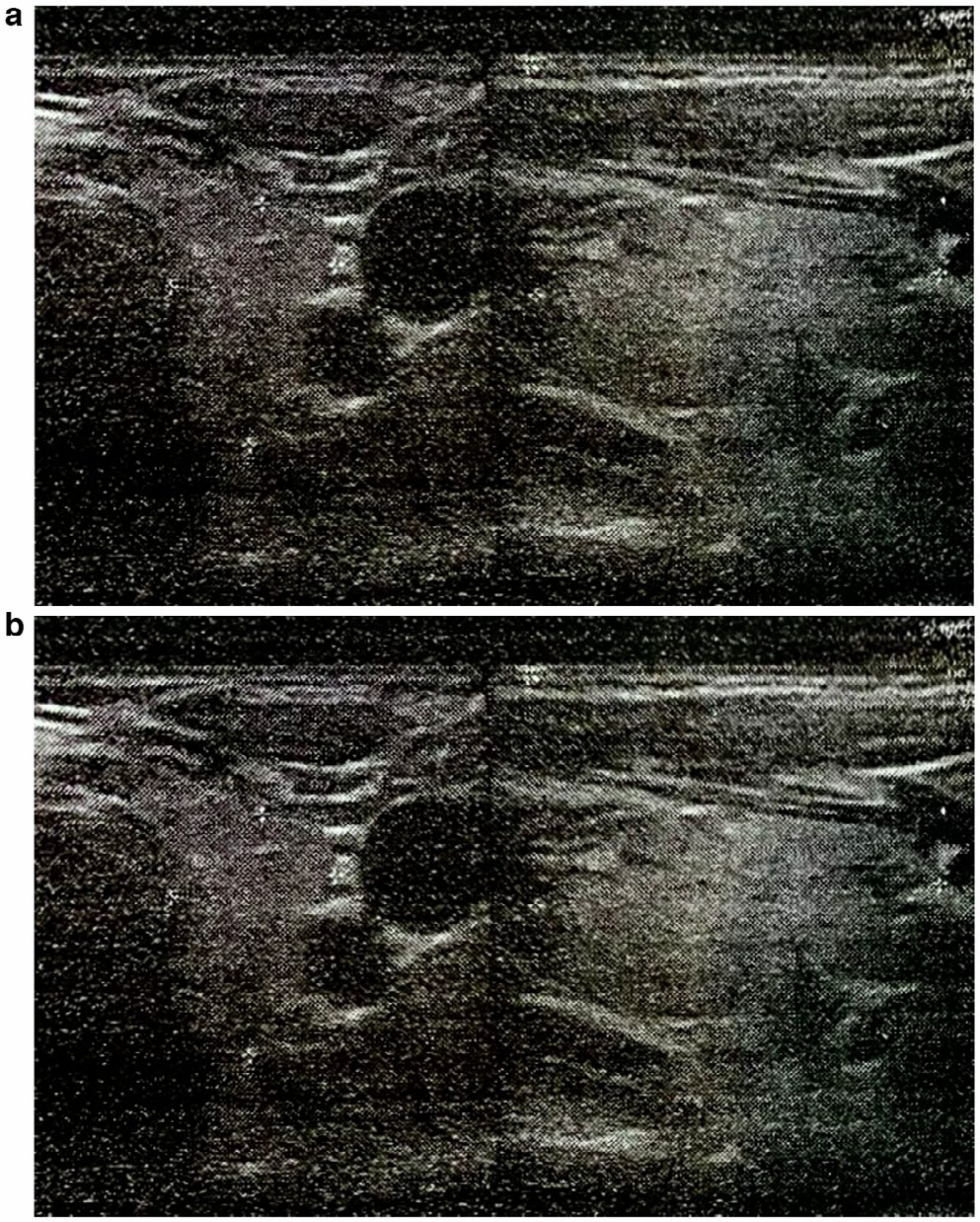
(a and b) Ultrasound showing bilateral lymphadenopathy.

The prominent symptoms of the case were constitutional symptoms consistent with many diseases, and the only specific symptoms were cervical lymphadenopathy and splenomegaly. Similar presentations can be seen in patients with lymphoma, acute HIV infection, toxoplasmosis, and brucellosis, for which our patient was screened and tested. The patient's complete blood count (CBC) showed a white blood cell (WBC) count of 17,900, with 75% lymphocytic predominance, indicating lymphocytosis (Table 1). Atypical lymphocytes were also seen. Cervical ultrasound showed reactive type (fatty‐centered) bilateral lymphadenopathy with the most significant node measuring 31 × 10 mm on the left (segment IIA) and 26 × 6 mm on the right side (segment VA) (Figure 1). The results of the blood tests showed significantly elevated LDH at 695 compared to the normal range of 135–225 for the patient. Also, IgG against EBV was 1.75, IgM against EBV was 2.66, both of which were strongly positive, and the Mono‐spot test was weakly positive. CRP and ESR were normal, and the tests for CMV, HIV, HCV, HBsAg, toxoplasmosis, malaria, borrelia, and brucellosis were negative (Table 2). These tests confirmed the diagnosis of IM.

**TABLE 1:** Complete blood count (CBC) and differential done by the laser scattering method.

Parameter	Result	Units	Reference range
WBC	17.9	× 10 ^ ^9^/L	3.5–10
RBC	5.28	× 10 ^ ^12^/L	4.5–6
Hemoglobin	15.5	g/dl	14–18
Hematocrit	46.1	%	39.7–52.2
Platelets	216	× 10 ^ ^9^/L	150–450
Neutrophils	19	%	
Lymphocytes	75	%	
Monocytes	5	%	
Eosinophils	1	%	
Total	100	%	

**TABLE 2:** Blood tests showing positive Mono‐spot test and elevated EBV IgM, IgG, and LDH.

Test	Result	Units	Reference range
Lactate dehydrogenase, serum	695 (high)	U/L	135–225
HIV‐1/−2 Ab, p24 Ag (CLIA)	Negative (0.31)	Index	Negative: < 1.0, reactive: ≥ 1.0
HBs Ag, serum (CLIA)	Negative (0.22)	Index	Negative: < 1.0, reactive: ≥ 1.0
HBs Ab, serum (CLIA)	19.43	IU/L	Negative: < 10.0, positive: ≥ 10.0
HCV Ab, serum (CLIA)	Negative (0.09)	Index	Negative: < 1.0, reactive: ≥ 1.0
Toxoplasma Ab, IgG, serum (CLIA)	0.60	IU/ml	Non‐Reactive: < 1.6, reactive: ≥ 3.0, equivocal: 1.6–3.0
Toxoplasma Ab, IgM, serum (CLIA)	Negative (0.09)	Ratio	Negative: < 0.83, equivocal: 0.83–1.0, reactive: > 1.0
EBV Ab, VCA IgG (CLIA)	1.75	Index	Negative: < 0.75, equivocal: 0.75–1.0, reactive: ≥ 1.0
EBV Ab, VCA IgM (CLIA)	2.66 (reactive)	Index	Negative: < 0.5, equivocal: 0.5–1.0, reactive: ≥ 1.0
C‐reactive protein (CRP), serum	6.32	mg/L	Negative: Up to 8.0
Wright, serum	Negative	Titer	Up to 1/80
Coombs wright, serum	Negative	Titer	Up to 1/80
2ME, serum	Negative	Titer	Up to 1/80
Mono‐spot test (Mononucleosis screen)	Weakly positive	—	Negative
Cytomegalovirus DNA PCR	Undetectable	—	Undetectable

## 4 Conclusion/Results (Outcome/Follow‐Up)

During the acute phase, the patient experienced transient constitutional symptoms, including fatigue and episodic sweats, which gradually resolved with conservative management. No progression of symptoms or development of complications was observed. The patient was followed up for 1 month and achieved complete clinical recovery. At 6‐month follow‐up, no relapse or delayed complications were identified.

## 5 Discussion

IM is not considered a rare disease; however, since most cases are treated as outpatients, there appears to be a scientific gap in reporting atypical cases, and most reported cases involve splenic ruptures or infarcts [9, 10, 11]. Identifying atypical cases of this disease is the main key challenge for medical practitioners today [12]. Previous systematic reviews have identified sore throat as the most common and sensitive clinical feature of IM [13, 14]. Therefore, a case presenting without a sore throat is not considered a possible case of IM; hence, it is investigated further. The present case presented with nonspecific constitutional symptoms, along with specific symptoms like cervical lymphadenopathy and splenomegaly. This presentation was consistent with lymphoma, acute HIV infection, toxoplasmosis, and brucellosis, for which our patient was screened and tested. In the absence of typical oropharyngeal symptoms, the clinical presentation may closely resemble malignant or systemic conditions, potentially altering the diagnostic trajectory [15]. Atypical presentations of IM that closely mimic malignant disease have been well documented in the literature. In a reported case of EBV‐associated IM involving the nasopharynx, histopathologic examination demonstrated atypical T cell proliferation that was initially interpreted as malignant T cell lymphoma, highlighting the potential for significant diagnostic error even after tissue biopsy [16]. In a larger clinicopathologic series, Louissaint et al. [17] evaluated 18 cases of acute IM that were submitted for consultation with an initial diagnosis of lymphoma. In these cases, polymorphous immunoblastic infiltrates and Reed‐Sternberg‐like cells led to frequent misclassification as malignant lymphoma before IM was correctly identified. Similar diagnostic challenges have also been reported in patients presenting with fever of unknown origin and lymphadenopathy, in whom IM mimicked lymphoma and prompted extensive diagnostic evaluations [5, 18]. An important and often underrecognized variant of IM is the so‐called “typhoidal mononucleosis,” characterized predominantly by prolonged fever and systemic constitutional symptoms with minimal or absent pharyngitis. In such presentations, the clinical picture may resemble typhoid fever or other systemic infectious or malignant conditions, leading to diagnostic uncertainty. Cunha et al. [5] described cases of EBV‐associated IM presenting as fever of unknown origin with a typhoidal pattern, initially raising concern for lymphoma or other serious diseases before the correct diagnosis was established. Recognition of this variant is clinically relevant, as it reinforces the need to consider IM even when classical oropharyngeal manifestations are not prominent. In comparison, the present case demonstrates that even in the absence of overt histopathologic atypia, atypical IM may clinically resemble malignancy, reinforcing the importance of early consideration of IM to avoid unnecessary invasive and costly investigations. Such diagnostic pathways can impose significant physical, emotional, and financial burdens on patients and health care systems [5]. From a clinical decision‐making perspective, this case emphasizes the importance of prioritizing common infectious etiologies early in the evaluation of patients presenting with fever and lymphadenopathy, even when classical features are absent. Incorporating EBV serology into the initial diagnostic workup may help prevent premature escalation toward invasive investigations and allow for a more targeted, cost‐effective, and patient‐centered diagnostic approach. Whereas IM can easily be diagnosed with a simple blood draw testing antibodies for EBV or polymerase chain reaction (PCR) for cytomegalovirus (CMV) for IM‐like features.

In patients suspected of IM who present without pharyngitis, a structured clinical evaluation is recommended. Initial assessment should focus on constitutional symptoms, lymphadenopathy, and splenomegaly, followed by basic laboratory investigations such as complete blood count with differential and peripheral smear. When lymphocytosis or atypical lymphocytes are present, EBV serology should be considered early.

This case underscores the importance of including atypical IM in the differential diagnosis of patients presenting with fever and lymphadenopathy, as failure to do so may result in misdiagnosis and unnecessary diagnostic escalation toward invasive and costly investigations.

## Author Contributions

**Mohammad Moini:** conceptualization, data curation, methodology, resources, writing – original draft, writing – review and editing. **Shiva Sheibani:** methodology, writing – original draft, writing – review and editing. **Matin Ghazizadeh:** conceptualization, supervision, writing – review and editing.

## Funding

The authors have nothing to report.

## Disclosure

The authors have nothing to report.

## Conflicts of Interest

The authors declare no conflicts of interest.

## Data Availability

Data available on request due to privacy/ethical restrictions.
